# Novel Common Integration Sites Targeted by Mouse Mammary Tumor Virus Insertion in Mammary Tumors Have Oncogenic Activity

**DOI:** 10.1371/journal.pone.0027425

**Published:** 2011-11-07

**Authors:** Hyoung H. Kim, A. Pieter J. van den Heuvel, John W. Schmidt, Susan R. Ross

**Affiliations:** Department of Microbiology/Abramson Cancer Center, Perelman School of Medicine, University of Pennsylvania, Philadelphia, Pennsylvania, United States of America; Institut Pasteur, France

## Abstract

Non-acute transforming retroviruses like mouse mammary tumor virus (MMTV) cause cancer, at least in part, through integration near cellular genes involved in growth control, thereby de-regulating their expression. It is well-established that MMTV commonly integrates near and activates expression of members of the *Wnt* and *Fgf* pathways in mammary tumors. However, there are a significant number of tumors for which the proviral integration sites have not been identified. Here, we used high through-put screening to identify common integration sites (CISs) in MMTV-induced tumors from C3H/HeN and BALB/c mice. As expected, members of both the *Wnt* and *Fgf* families were identified in this screen. In addition, a number of novel CISs were found, including *Tcf7l2*, *Antxr1*/*Tem8*, and *Arhgap18*. We show here that expression of these three putative oncogenes in normal murine mammary gland cells altered their growth kinetics and caused their morphological transformation when grown in three dimensional cultures. Additionally, expression of *Tcf7l2* and *Antxr1*/*Tem8* sensitized cells to exogenous WNT ligand. As *Tcf7l2*, *Antxr1*/*Tem8*, and *Arhgap18* have been associated with human breast and other cancers, these data demonstrate that MMTV-induced insertional mutation remains an important means for identifying genes involved in breast cancer.

## Introduction

MMTV-induced mammary tumorigenesis in mice has long been used as a model for the study of human breast cancer. MMTV-induced transformation is mediated by proviral integration near cellular oncogenes in mammary epithelial cells, and a number of different common integration sites (CISs) have been implicated in this process [1]. In particular, members of the *Wnt* and *Fgf* family are frequently targeted by MMTV, although a number of additional CISs have also been identified, particularly in infected feral mice such as Czech II or M. spretus [2], [3], [4], [5]. In addition to insertional activation of cellular oncogenes, the MMTV envelope glycoprotein participates in oncogenic transformation of mammary epithelial cells, through the action of an Env-encoded immunoreceptor tyrosine activation motif (ITAM) [6], [7]. Tumors derived from mice infected with MMTV containing ITAM-mutant envelopes showed an altered pattern of *Wnt* and *Fgf* activation compared to mice infected with wild-type MMTV [7], indicating that different oncogenes might be required for transformation in the absence of ITAM-mediated signaling.

A number of groups have used high throughput analysis to identify retroviral integration sites in murine leukemia virus (MLV)-transduced cells and after MLV and MMTV proviral, as well as retrotransposon insertion in tumors as a means of identifying novel oncogenes [2], [4], [8], [9], [10], [11], [12]. Many of these studies have been compiled into a database termed the Retrovirus-Tagged Cancer Gene Database (RTCGD) [13]. However, most of the novel hits deposited in the RTCGD have not been validated in biological assays.

Here, we used a high throughput approach to identify additional CISs in MMTV-induced mammary tumors and found several novel MMTV target genes including *Arhgap18*, *Tcf7l2*, *Prkaca* and *Antxr1*/*Tem8* (called *Antxr1* from here-on). We show that transduction of expression vectors bearing *Arhgap18*, *Tcf7l2* and *Antxr1* into normal murine mammary cells resulted in altered growth properties and caused morphological transformation. Thus, these genes likely participate in oncogenesis *in vivo*.

## Results

### Identification of MMTV CISs

We used linker-mediated PCR to clone integration sites in the MMTV-induced tumors [14] (see Materials and Methods). The tumors were derived from C3H/HeN mice infected with MMTV(C3H) and BALB/c mice infected with MMTV(HP) or MMTV(Y1Y2) [15], all of which acquired the virus neonatally through milk-borne transmission. MMTV(HP) is a MMTV molecular clone consisting of the 5′ half of an endogenous MMTV (*Mtv-*1) and the 3′ half of the MMTV(C3H) [16]. MMTV(Y1Y2) was derived from MMTV(HP) by introducing point mutations into the *Env* ITAM motif that diminished its transforming ability [6], [7]. Tumor DNA was isolated from tumors that developed at single sites in individual mice. The tumors had from around 10 to 100 integrations of the MMTV provirus (not shown).

We screened and sequenced over 1500 integration sites from 33 BALB/c (17 MMTV(HP), 16 MMTV(Y1Y2)) and 26 C3H/HeN tumors and were able to place 947 integration sites in the genome, of which 347 were unique. The remainder either showed matches to multiple locations, did not yield a high-quality match to the mouse genome (e.g. integration events in regions of the mouse genome not in the sequence database) or were excluded as low-quality sequence reads (e.g. sequences too short to determine a unique placement).

Many of the tumors showed the standard integrations into the *Wnt1* and *Fgf3* genes, as well as into other *Wnt* (*Wnt3*, *Wnt3a*) and *Fgf* (Fgf8) family members (Table 1), as has been previously reported [1], [4]. We not only found integrations into *Wnt* genes but also in genes in the *Wnt* signaling pathway (*Lrp5*, *Parp1*, *Ror1*, *Tcf7l2*, *Foxl1*) as well as targets of *Wnt* signaling (*Cdk6*, *Ccnd1*, *Dlk1*, *Eda2r*, *Epha6*, *Nfatc3*) (Table 1). We also found CISs near *FgfR3*, *FgfR2* and *FgfR1* in different tumors, indicating that MMTV may be able to cause tumors by increasing expression of either the ligand or receptor for this family of growth factor molecules (Table 1).

**Table 1:** Selected CISs found in MMTV-induced mammary tumors.

						RTCGD Database		
Gene	Insertion	IS dist. *	Expression **	BALB +	C3H +	Virus	Type	HBC #
**Wnt pathway**								
*Antxr1/Tem8*	intron (1)	26, 108	+	1	1			LBC, IBC
*Eda2r*		−6	-	0	1			IBC
*Epha6*	intron	23	-	0	1			
*Foxl1*		−71	+	1	0	MMTV	Mammary	ILC, DC
*Hhip*	intron	0.8	-	0	1			
*Jag1*	intron	11	++	0	1			
*Lrp5*	intron	39	-	0	1			
*Nfatc3*	intron	25	-	0	1	AKV	B cell	
*Parp1*		38	-	1	0			LBC, IBC, DC
*Ror1*	intron	60	-	0	1			
*Tcf7l2*		14, −66	++++	0	2			
*Wnt1/ Wnt10b*			-	6	10	MMTV	Mammary	
*Wnt3/Wnt9b*	intron		-	5	0	MMTV	Mammary	IDC
*Wnt3a/Wnt9a*	exon (1)		-	4	1	MMTV	Mammary	
**Fgf pathway**								
*Fgf3/Fgf4*			-	11	8	MMTV	Mammary	
*Fgf8*			-	2	2	MMTV	Mammary	
*Fgfr1*			-	0	2			
*Fgfr2*	Intron (2)		-	2	2			IDC, DC
*Fgfr3*			-	0	1	M-MLV, AKV	B, T cell	
**Other**								
*Arhgap18*		5, 0.4	+	0	2			IBC
*Bai3*	Intron (1)	91 (I), 126	-	0	2			
*Igf2/Ins2*		1, 6, 9	+	2	1	MMTV	Mammary	
*Pdgfra*	Intron (1)	2 (I), 1	-	0	2			
*Prkaca*	Intron (1)	6 (I), 30	+++	0	2			

*Distance in kb from start of transcription for CIS other than *Wnt* or *Fgf* genes.

**expression in normal mammary tissue (data from BioGPS).

+ number of BALB and C3H tumors with this CIS.

# data from Oncomine database. Abbreviations: HBC, human breast cancer; LBC, lobular breast carcinoma; IBC, invasive breast carcinoma; ILC, invasive lobular carcinoma; DBC ductal carcinoma.

In addition to genes in the *Wnt*, *Fgf* and *Notch* pathways, there were a number of sites that were identified as novel CISs in 2 (*Bai*3, *Arhgap18*, *Pdgfra* and *Prkaca)* or more (*Igf2*) independent tumors, (Tables 1 and 2). We compared our MMTV integration sites with those in the RTCGD and found about >70 IS in common between the 2 databases; a representative sample of some common sites is shown in Table 1. Similarly, we have compared our integration sites to those reported in other MMTV screens and found overlap in genes such as *Igf2, FgfR2* and *Pdgfra*, as well as the *Wnt* and *Fgf* family members [4], [17].

**Table 2:** Integration sites in tumors with novel CIS.

Tumor	CIS	Copy #	other insertions in tumor
C3H C	*Tcf7l2*	105	*Serpinb8,Lhx4,Cdca1,Nmt2,Qscn6l1,Eya2,Zfp217,Tpd52,Slc44a3,Rbm35a,Fgfr3,Aff1,Antxr1,Prkaca,Bai3*,*Cdh8,Pgr,Igsf4a,Hcrtr2,Plod2,Ctdspl,Hsf2,Slc39a11,Tnfsf11,Krt85,Casr,Safb2,Cdh2,Gypc,Atp8b1,Gm815,Cyp2c29,Usp9x,Actrt1,Pgr15l,Eda2r,E230019M04Rik,Gpr64,BC023488,E130016E03Rik,AL033314,4930544G11Rik,LOC432436,LOC628586,2310022M17Rik,6030413G23Rik,4921528I01Rik,*
C3H BF		9	*Atp9a,Sfrs8,A430010J10Rik,Wnt1,Prodh,Tcf7l2*
BALB 5	*Antxr1*	ND	*Kcne4,Htra1,BC035537,Wnt3,Calml3,2410141K09Rik*
C3H C			*See above*
C3H A	*Arhgap18*	10	*1700001O22Rik,Dhrs9,Eltd1,F730047E07Rik,Rod1,2810055G22Rik,Pon1,Cnga4,Mgmt,Fgf3,Tpcn2,Hhip,Bai3,1110059P08Rik,Btbd11,C630004H02Rik,Fbxo33,Drd1a,Ubqln1,Emb,Sh2d4b,Azin1,Olfr161, Epha6,Gbe1,Dnahc8,Tbn*
C3H BN		ND	*4933421I07Rik, Ins2*
C3H A	*Bai3*		See above
C3H C			See above
C3H C	*Prkaca*		See above
C3H P		10	*Rab2,Coro2a,D030010E02Rik,Fgf3,Nfatc3,Lpin3*
C3H F	*Pdgfra*	17	*Scg2,Cxxc4,Cacna2d1,Prss23,Foxl1,Mark3,Lrrc16,Wnt10b,Vbp1*
C3H D		12	*Gpr88,AW049829,Fgfr2,Fgf3,Tbc1d9,Six6os1,Hes1,Htr2c*
BALB 45	*Igf2*	ND	*1700011F14Rik*
BALB 11		12	*Cxcl5*
C3H BN			*4933421I07Rik, Arhgap18*

All of the identified integration sites in tumors with novel CIS (see Table I) are listed. Additional CIS found in the tumors are underlined.

### Expression of CISs in tumors

We used RT-PCR to analyze some of the tumors for expression of the MMTV target genes *Wnt1*, *Wnt10b*, *Wnt3*, *Wnt3a*, *Fgf3*, *Fgf4* and *Fgf8* and correlated this expression with the CIS analysis (Table 3). We identified insertions in about 70% of the tumors that expressed *Wnt1* and *Fgf*3, indicating that the cloned integration sites were representative. Importantly, for *Wnt1* and *Fgf3*, with the exception of 3 tumors with an insertion near *Wnt1* and 2 tumors with an insertion near *Fgf3*, the tumors containing these CISs expressed these genes. This demonstrates that for the *Wnt* and *Fgf* genes, expression correlates well with integration.

**Table 3:** RT-PCR analysis of BALB/c (B) and C3H (C) tumors.

Tumor	*Wnt* 1	*Wnt* 10	*Wnt* 3A	*Wnt* 3	*Fgf* 3	*Fgf* 4	*Fgf* 8	CIS
**MMTV(HP)**								
**B3**	+	+	-	-	+	-	+	*Fgf3*
**B5**	-	+	-	+	-	+	+	*Wnt3*
**B6**	-	-	-	+	+	+	+	*Wnt3*
**B20**	-	+	-	-	+	+	-	*Wnt1, Fgf3*
**B21**	+	+	+	-	+	-	+	*Wnt1, Fgf3*
**B22**	-	-	-	+	+	-	+	*Fgf3*
**B23**	+	+	+	-	+	+	-	*Wnt1, Fgf3*
**B26**	-	-	-	+	+	-	+	*Fgf3*
**B27**	+	+	-	-	+	-	+	*Fgf3*
**B28**	-	+	+	-	+	-	-	*Wnt1, Fgf3*
**B30**	-	+	+	+	+	-	-	*Wnt3*
**B31**	-	-	+	-	+	-	+	*Wnt3a, Fgf3*
**B41**	-	+	+	-	-	-	+	*Wnt3a*
**MMTV(Y1Y2)**								
**B7**	-	-	-	+	-	-	-	*Wnt3*
**B10**	-	+	-	+	-	-	+	*Fgf8*
**B12**	+	+	-	-	-	-	+	*Fgf8*
**B17**	-	+	+	-	+	-	+	*Wnt3a*
**B32**	-	+	+	-	-	-	+	*Wnt3a*
**B33**	+	+	+	-	-	-	+	*Wnt1*
**B39**	-	+	+	-	+	-	+	*Fgf3*
**MMTV(C3H)**								
**C-A**	-	+	-	**ND**	-	**ND**	-	*Fgf3*
**C-B**	+	+	-	+	+	-	+	*Wnt1, Fgf3*
**C-C**	-	-	-	-	+	+	+	none
**C-D**	+	+	-	-	+	+	+	*Fgf3*
**C-F**	+	+	-	-	+	**ND**	+	*Wnt1, Fgf3*
**C-G**	+	+	-	-	+	+	+	*Wnt1*
**C-H**	+	+	-	-	-	+	+	*Wnt1*
**C-K**	+	+	-	-	-	**ND**	+	*Wnt1*
**C-P**	+	+	-	+	-	-	+	*Fgf3*
**C-W**	-	-	-	-	+	+	-	*Wnt1, Fgf3*
**C-AC**	-	-	+	-	+	-	+	*Wnt3a*
**C-AE**	+	+	+	-	+	+	+	*Fgf3*
**C-BF**	+	+	-	-	-	-	+	*Wnt1*
**BN**	+	**ND**	-	-	+	**ND**	**ND**	none

CIS: *Wnt* or *Fgf* integration identified by cloning. Expression: +, expression of oncogene; -, no expression of oncogene; ND, not done.

Because *Wnt1*/*Wnt10b*, *Wnt3*/*Wnt9b*, *Wnt3a*/*Wnt9a* and *Fgf3*/*Fgf4* are all close to each other in the genome, respectively, the integration site alone would not indicate which genes are activated. Many of the tumors with insertions at the *Fgf3*/*Fgf4* locus did not express *Fgf4*, indicating that activation of *Fgf3* is more important for MMTV-induced tumorigenesis. There was also only ∼40% correlation between expression and integration for the *Wnt3* and *Wnt3a* genes (Table 3). Expression of an gene in a tumor in which integration into that gene was not detected may reflect incomplete identification of all the integration sites in a given tumor or that expression of these genes occurs downstream of other integration events or changes to the cells leading to transformation. This appears to be the case for *Fgf8*, where only 2/26 tumors that showed increased expression were identified with integrations in this gene.

**Figure 1:**
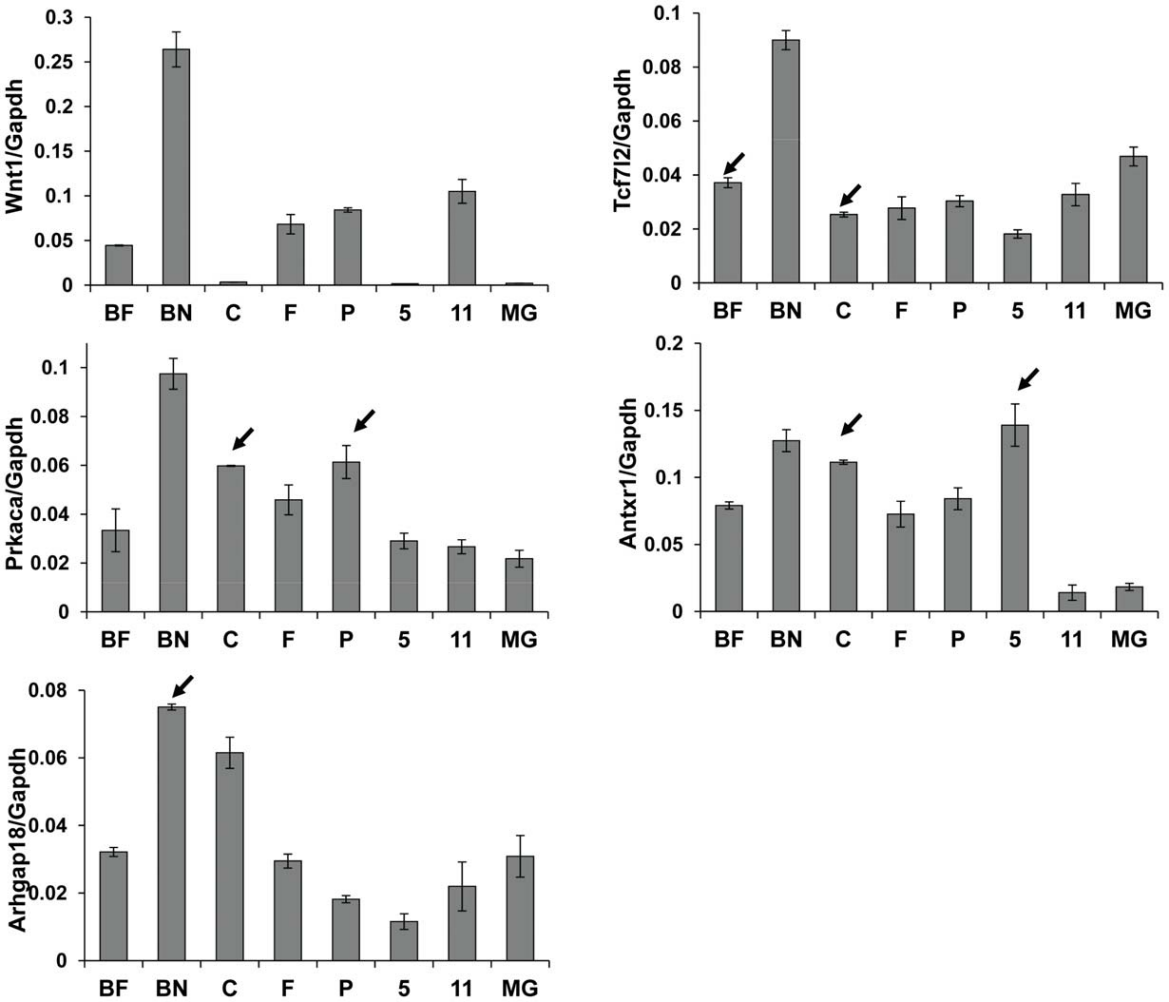
Expression of selected CIS genes in MMTV-induced mammary tumors. RNA was extracted from eight MMTV-induced mammary tumors; 5 from C3H mice (BF, BN, C, F, P) and 2 from BALB/c mice (5, 11). Normal mammary gland (MG) was obtained from a 4 month old virgin mouse and used as a control. Expression of selected CIS genes (*Wnt1*, *Prkaca*, *Arhgap18*, *Tcf7l2*, *Antxr1*) were measured by qRT-PCR. GAPDH was used as the endogenous control gene. The relative levels of the gene specific PCR product were normalized to GAPDH. All error bars represent standard deviations. Arrows indicate tumors harboring the corresponding CIS.

We also examined expression of *Antxr1*, *Tcf7l2, Arhgap18* and *Prkaca* by RT-qPCR (Fig. 1); *Wnt1*, whose expression correlates well with integration (Table 1), was used as a control. Four tumors including two tumors with insertions near *Prkaca* (C and P) showed higher expression of the *Prkaca* transcript compared to normal mammary gland. Two tumors including one tumor with insertion near *Arhgap18* (BN) showed higher expression of this gene. *Antxr1* RNA levels were high in all tumors except one compared to normal mammary gland; the two tumors with insertions near *Antxr1* (C and 5) were among those that most highly expressed this gene. In contrast, the two tumors with insertions near *Tcf7l2* did not show increased expression. *Tcf7l2* is highly expressed in normal mammary tissue, as well as adipose tissue (Fig. 1 and Table 1; [18]). Thus, it is possible that comparison of expression in mammary tumors, which are mostly composed of epithelial cells, to that in normal mammary gland, which is a mixture of epithelial cells, connective tissue and adipocytes as well as low levels of hematopoietic cells, may mask insertion site effects on expression. Alternatively, integration into this locus could alter gene expression at early times in the transformation process, before the tumors are harvested.

### Expression of *Arhgap18*, *Tcf7l2* and *Antxr1* in normal mammary epithelial cells enhances cell growth and proliferation

**Figure 2:**
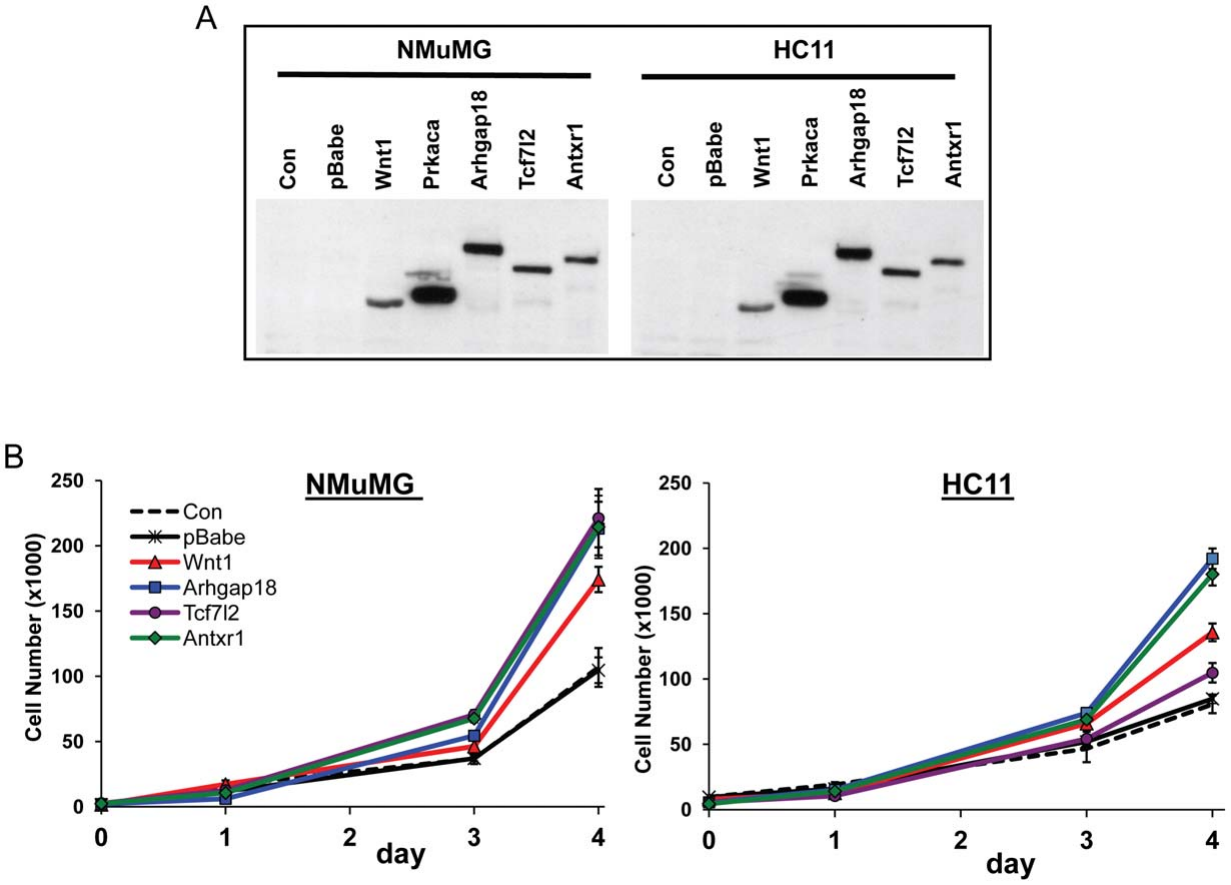
Expression of target CIS proteins in transduced mammary epithelial cells and enhanced cell growth. The pBabe retroviral constructs containing the cDNAs of *Wnt1*, *Arhgap18*, *Tcf7l2*, and *Antxr1* were transduced into NMuMG and HC11 mouse mammary cells. A) Cell extracts from the different transduced cells were subjected to western blot analysis using anti-HA antibodies. B) Cells stably transduced with CIS genes were plated in 96-well plates and grown for 4 days. Cell growth was monitored by the MTT assay (Materials and Methods). Data represents mean values with the SE (n = 5). Data were analyzed by two way ANOVA analysis with Dunnett's post hoc test. At day 4, all CIS-transduced NMuMG cells and all but the *Tcf7l2*-transduced HC11 cells showed significant differences from control cells (p≤0.01).

Most screens to identify CISs in retrovirus-induced tumors have not functionally tested whether the genes at the insertion site perturb the growth properties of cells. Normal mammary epithelial cells have distinctive growth properties and moreover, can be grown in 3-dimensional cultures where they form structures resembling mammary gland alveolar end buds. To determine whether expression of novel CISs affect the growth properties of normal mammary epithelial cells, *Tcf7l2*, *Arhgap18* and *Antxr1* were HA-tagged, cloned into a retroviral vector and transduced into two different normal mammary epithelial cell lines, NMuMG (normal murine mammary gland from the NAMRU mouse) and HC11 (BALB/c mouse). HC11 cells have mutations in p53 believed to contribute to their immortalization [19]. Both parental cell lines differentiate into alveolar end buds when grown in three-dimensional cell culture [6], [20]. Protein expression in pools of transduced cells was confirmed by immunoblotting against the anti-HA tag (Fig. 2A). We also cloned the known MMTV CIS *Wnt1* oncogene into the retroviral vector and used this construct as a positive control (Fig. 2A); empty vector (pBabe)-transduced cells served as negative controls.

First, we monitored cell growth of the different transduced cell lines. NMuMG cells transduced with all 4 genes (*Wnt1*, *Arhgap18*, *Tcf7l2*, *Antxr1*) grew more rapidly compared to pBabe- or untransduced cells (Fig. 2B). In contrast, while the *Wnt1*-, *Arhgap1*- and *Antxr1*-transduced HC11 cells all had enhanced growth, the *Tcf7l2*-transduced cells were not significantly different from the controls (Fig. 2B). This difference could be due to the genetic backgrounds of the 2 different epithelial cell lines (i.e. the mutated p53 gene in HC11 cells) and was not investigated further.

**Figure 3:**
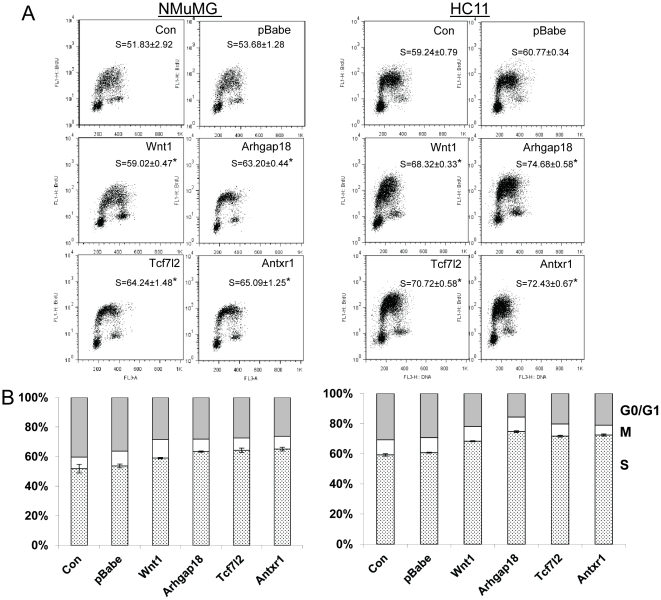
Expression of CIS genes in transduced mammary epithelial cells enhances cell proliferation. A) FACS plots for different transduced cells. S, percentage of cells in S phase averaged from 3 independent samples. *p≤0.05, determined by Student's t test. B) Bar graphs show the percentage of cells in G0/G1 (shaded), M (open) and S (stippled phase). Shown is the average of 3 experiments; error bars show the SE.

Next we analyzed cell proliferation in the different transduced cells using BrdU incorporation. In the NMuMG- and HC11-transduced cells expressing all 3 novel CISs as well as *Wnt1*, the percentage of cells in G1/G0 was decreased and those in S phase were significantly increased compared to the parental or pBabe-transduced cells (Fig. 3). Taken together with the cell growth analysis, these data indicate that the 3 novel CISs identified in our screen all increased cell proliferation. Importantly, the CIS- and *Wnt1*- transduced cells showed similar growth properties, indicating that these genes had similar oncogenic activities.

### Increased expression of CIS genes triggers morphological transformation of mammary epithelial cells

Mammary epithelial cells recapitulate several aspects of mammary organogenesis when grown on an exogenous basement membrane such as laminin-rich matrigel, including the formation of polarized, acinar-like spheroids with hollow lumens similar to terminal end buds and the basal deposition of basement components [21]. Such cultures can also be used to distinguish epithelial cells from normal and tumorigenic tissue and to dissect the mechanisms by which known oncogenes perturb normal mammary gland growth and development. For example, we previously showed that expression of the MMTV envelope protein in normal mammary epithelial cells caused their morphological transformation when grown in three-dimensional but not two-dimensional cultures [6]. We next tested the transduced HC11 and NMuMG cells in matrigel cultures to determine whether expression of *Wnt1*, *Arhgap18*, *Tcf7l2* or *Antxr1* caused morphological transformation of normal mammary epithelial cells.

**Figure 4:**
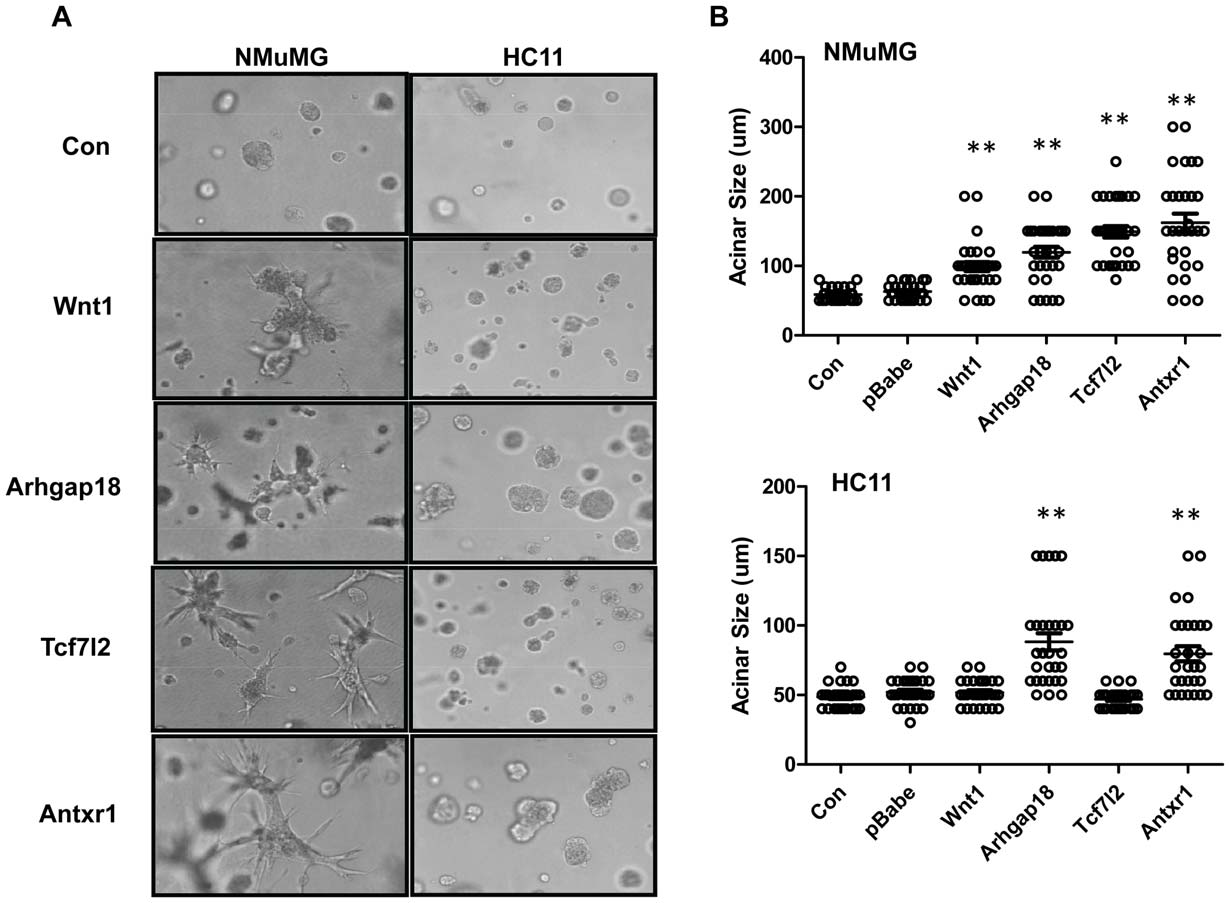
Increased expression of CIS genes causes morphological transformation of mammary epithelial cells in 3D culture. A) NMuMG or HC11 cells transduced with the different CIS genes or empty vector (control) were grown in 8 well chamber slides with matrigel. Images were captured at day 10 (200X magnification). B) Acinar size was measured on day 10 of growth in matrigel. Acini with diameters over 50 µm were scored (n = 30 for each cell line). **p≤0.01, determined by Student's t-test.

When grown in matrigel cultures, the control cells developed into small, spherical structures at day 6 (not shown); the NMuMG and HC11 cells structure formed acini-like, hollow lumens at day 10, with 50 µm and 70-80 µm diameter acini, respectively (Fig. 4A). In contrast, by day 6 the transduced cells developed into larger asymmetric aggregates (not shown). By day 10 day, all the transduced NMuMG cells, as well as the *Arhgap18* and *Antxr1*-transduced HC11 cells, however, developed much larger, irregularly shaped structures, often with cells filling the luminal space, a characteristic of the early neoplastic transformation of breast epithelium (Fig. 4A and 4B). Neither *Wnt1* nor *Tcf7l2* over-expression in HC11 cells caused their morphological transformation. This is similar to what was seen in the cell proliferation assay, where the expression of the different putative oncogenes, particularly *Tcf7l2*, had a more pronounced effect on NMuMG cells (Fig. 3). Again, this differential effect of the CISs on NMuMG and HC11 cells is likely due to genetic differences between the two lines.

### The *Wnt* signaling pathway is altered in *Tcf7l2* and *Antxr1*-transduced cells

The *Wnt* signaling pathway is involved in the determination of cell and tissue polarity, stimulation of cell proliferation and differentiation, and adult tissue homeostasis [22], [23]. Since two of the CISs are known members of the *Wnt* signaling pathway (*Wnt1*, *Tcf7l2*) and *Antxr1* may act in the *Wnt* pathway [24], we next tested whether this pathway was activated in the transduced cells. The primary receptor for the *Wnt* protein is comprised of Frizzled (Fz) a seven transmembrane receptor family (10 members) and low density lipoprotein receptor-related protein (LRP) single transmembrane receptors, LRP5 or LRP6. In the absence of a *Wnt*-signal, cytoplasmic β-catenin is rapidly marked for degradation by the anaphase-promoting complex (APC). In contrast, when extracellular *Wnt* binds to surface receptors, the activity of the APC complex is inhibited and, consequently, stabilized β-catenin enters the nucleus, where it binds to transcription factors from the *Tcf/LEF* family and eventually induces the expression of numerous *Wnt* target genes including *c-Myc*, transferrin receptor 1 (*Tfr1*), *Axin1* and *CcndD1* [25].

**Figure 5:**
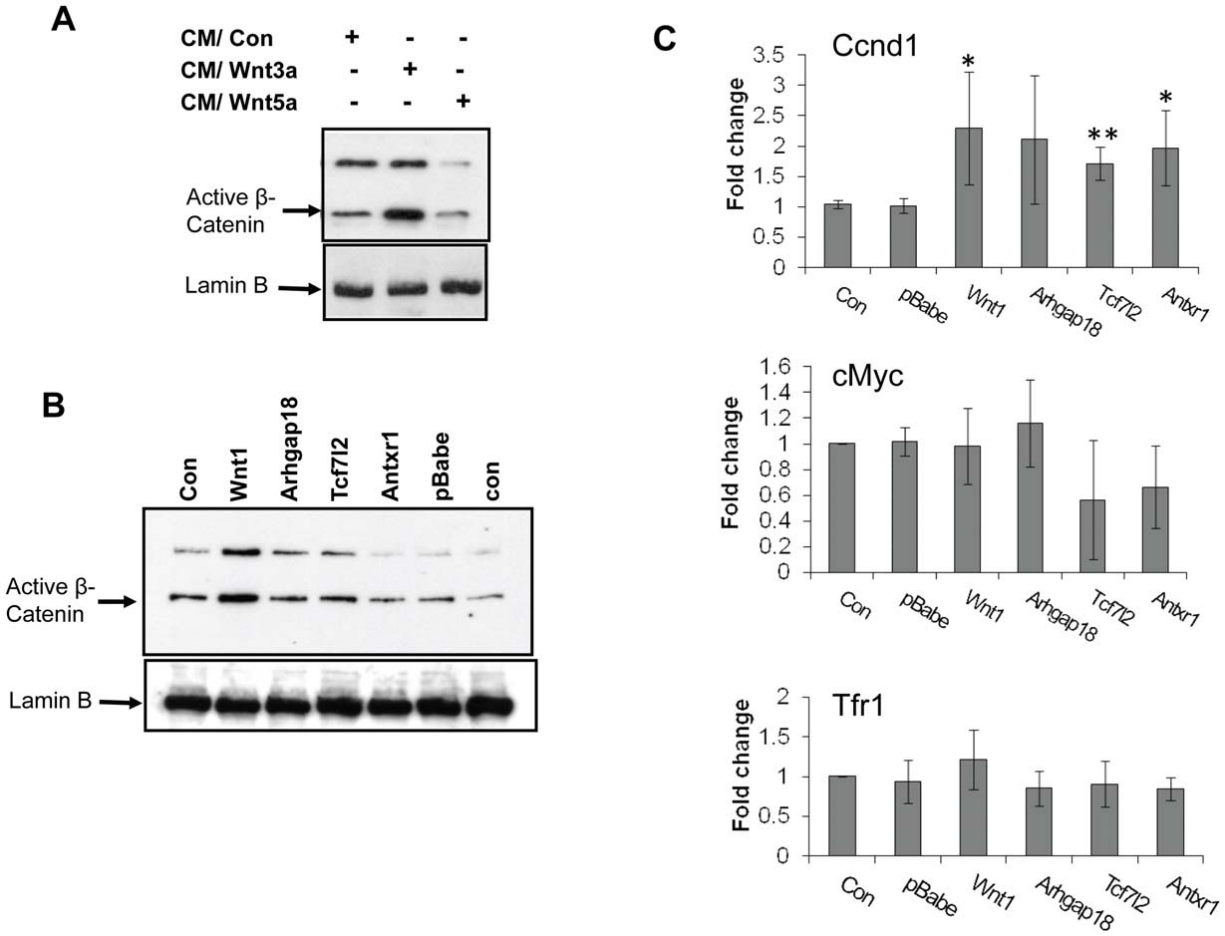
Basal WNT signaling pathway in the CIS-transduced cells. A) Untransduced NMuMG cells were incubated with 20% Wnt3a- or Wnt5a-containing media for 48 hr. Nuclear protein was extracted and active β-catenin levels were analyzed by immunobloting. Samples were also probed with the anti-lamin B antibody to verify equal loading. B) Nuclear extracts were obtained from NMuMG cells transduced with CIS genes and active β-catenin level was measured by immunobloting. Samples were also probed with the anti-lamin B antibody to verify equal loading. C) mRNA was extracted from NMuMG cells transduced with CIS genes and RT-qPCR was used to measure Ccnd1, Myc, and Tfr1 RNA levels. All values were normalized to GAPDH. Data are the means of three independent experiments with the SE values indicated by error bars. Data were analyzed by Student's t-test. *p≤0.05; **p≤0.01 versus the corresponding control value, as determined by Student's t-test.

To examine *Wnt* signaling, we first measured activated β-catenin levels in the transduced NMuMG cells. As a control, we treated untransduced NMuMG cells with WNT3A- or WNT5A-containing media (see Materials and Methods). WNT3A but not WNT5A treatment caused increased activated nuclear β-catenin, in agreement with the induction of the canonical Wnt signaling pathway by the former but not the latter (Fig. 5A). Increased activated nuclear β-catenin levels were seen in the Wnt1-transduced cells, but not in the *Arhgap18*-, Tcf7l2- or Antxr1- expressing cells (Fig. 5B).

**Figure 6:**
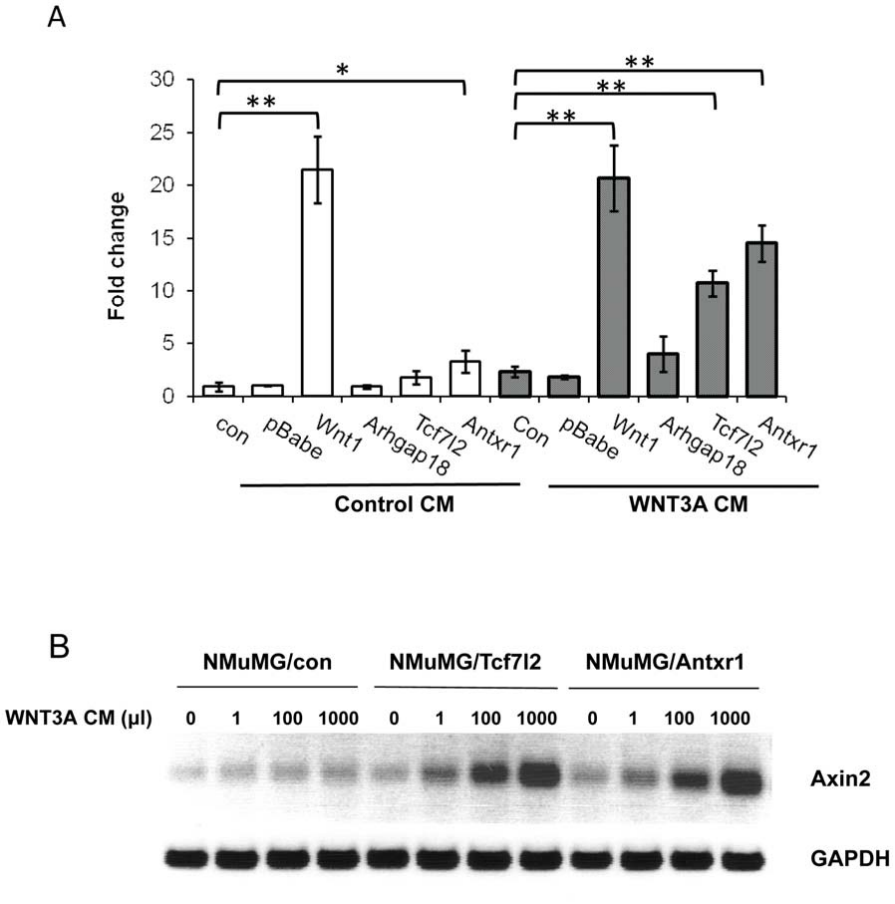
NMuMG cells transduced with *Tcf7l2* and *Antxr1* show enhanced response to an exogenous WNT signal. A) NMuMG cells stably transduced with the CIS genes were grown in control (Control CM) or WNT3A (WNT3A CM) conditioned media for 48 hr. The level of Axin2 was then examined by RT-qPCR. All values were normalized to GAPDH. Data are the means of three independent experiments with the SE values indicated by error bars. **p≤0.01, as determined by Student's t-test. B) *Tcf7l2* and Antxr1-expressing cells were incubated with increasing dose of WNT3A CM for 48 hr (1, 10, 100, 1000 · l in 10 ml media) and *Axin2* RNA levels were examined by RT-PCR. GAPDH was used as internal control.

Next, we examined the RNA levels of four *Wnt* signaling target genes, *Ccnd1*, c-*Myc*, *Tfr1* and *Axin2* in the CIS-transduced NMuMG cells. *Ccnd1*, *Myc* and *Tfr1* are the target genes for multiple signaling pathways. In contrast, *Axin2*, which is involved in the negative regulation of the β-catenin protein through destabilization, is believed to be specifically up-regulated by signaling through the canonical WNT/β-catenin pathway [26]. *Ccnd1* RNA levels were increased in Tcf7l2- and Antxr1- transduced cells as well as the *Wnt1*-expressing cells (Fig. 5C). However, the levels of *Myc and Tfr1* RNA were not significantly changed in any transduced cells (Fig. 5C). Basal *Axin*2 RNA levels were greatly increased in the *Wnt1*-transduced NMuMG cells (20-fold), and to a lesser extent in the *Tcf7l2* and *Antxr1*-expressing cells (2- and 3-fold, respectively) (Fig. 6A, control CM). These data suggest that over-expression of *Tcf7l2* and *Antxr1* in the normal mammary cell lines resulted in moderate activation of the WNT signaling pathway, but to a lesser extent than was seen in WNT1-expressing cells.

### *Tcf7l2* and *Antxr1*-transduced cells are responsive to exogenous WNT ligand

Although basal WNT signaling was not greatly changed in *Tcf7l2* and *Antxr1*-expressing cells, it was possible that these cells would be more responsive to exogenous WNT ligands, since both proteins are believed to function in the this pathway, one at the cell surface (ANTXR1) and the other in the nucleus (TCF7L2). To test this hypothesis, the cells were treated with WNT3A-conditioned media. While treatment of the *Wnt1*-expressing cells with exogenous WNT3A had no effect on *Axin2* RNA levels, presumably because of the high levels of WNT1 already made in these cells, treatment of the *Tcf7l2*- and *Antxr1*-transduced cells resulted in a large increase in *Axin2* RNA (Fig. 6A). The *Arhgap18*-transduced cells also showed no significant response to WNT3A, suggesting that this gene transforms mammary cells by a WNT-independent pathway. This increased responsiveness was a function of WNT3A in the media, since the increase in *Axin2* RNA levels in *Tcf7l2*- and *Antxr1*-expressing cells was proportional to the amount of exogenous WNT3A-conditioned media added to the cultures (Fig. 6B).

Thus, while activation of the WNT pathway was minimal in the *Tcf7l2*- or *Antxr1-*transduced cells, expression of either gene in normal mammary gland epithelial cells rendered them more sensitive to exogenous WNT stimulation (Fig. 6). This type of sensitization could also occur in tumor cells. For example, tumor C3H BF had *Wnt1* as a CIS in addition to *Tcf7l2*, while insertion of MMTV at *Antxr1* and *Wnt3* in tumor Balb 5 resulted in the expression of both CISs (Fig. 1 and Tables 2 and 3). Interestingly, no *Wnt* genes were found as CISs in tumor C (Tables 2 and 3). However, this tumor had integrations at both *Tcf7l2* and *Antxr1* which could potentially result in enhanced signaling by over-expression of 2 genes in the same WNT pathway.

## Discussion

Since its discovery as an oncogenic retrovirus, MMTV has provided one of the most useful models for understanding human breast cancer. Included in the major advances made in breast cancer research was the finding that MMTV induced mammary tumors through the action of virus-encoded transcriptional regulatory elements that activate cellular oncogene expression after proviral integration [27]. This finding has led to the development of more than 100 transgenic models of breast cancer in which the MMTV transcriptional regulatory region drives the expression of potential oncogenes [3]. Additionally, a number of cellular oncogenes, such as *Wnt1* and *Fgf3*, were first identified by identification of the CISs found in both wild type and transgenic MMTV-infected mice and later shown to be active in pathways relevant to the human disease.

Several of the CISs identified here, such as *Antxr1* and *Tcf7l2*, also function in the WNT signaling pathway. *Antxr1* was originally identified as a receptor for anthrax toxin, although more recent work has indicated that it is not the primary toxin receptor [28], [29]. This gene is also called *Tem8* (Tumor endothelial marker-8) because it is expressed during developmental angiogenesis and in tumor endothelium [30], [31]. It has recently been reported that ANTXR1 is a interacting partner of LRP6, the well known co-receptor of WNT signaling [32]. ANTXR1 appear to be required for the stabilization of LRP6; *Antxr1* knockdown caused down-regulation of LRP6, and inhibited WNT signaling [24]. Additionally, similar to the effects we described here, a recent study showed that ANTXR1 expression in HEK293 cells amplified β-catenin-dependent transcriptional activity in response to exogenous WNT [33]. ANTXR1/TEM8 also functions as an adhesion receptor and mediates actin-dependent spreading of cells on collagen [34]. The ability of ANTXR1 to interact with the extracellular matrix and with LRP6 raises the possible existence of a matrix-modulated mechanism for local control of WNT signaling [24]. Thus, in the case of MMTV, insertion of the virus at both the *Wnt* and *Antxr1* loci in a single tumor could potentiate growth of the transformed cells.

The *Tcf*/*Lef* transcription factors are the most downstream components of the *Wnt* signaling cascade. TCF proteins do not function as classical transcription factors, in that DNA binding alone is not sufficient to cause transcriptional activation. Promoter activation is only achieved after TCF complexes with β-catenin to generate a functional transcription factor [35]. TCF proteins can also interact with co-repressors to down-modulate WNT target gene expression in the absence of WNT signaling [35]. Indeed, TCF7L2 acts as a tumor suppressor in colon cell proliferation and tumorigenesis [36], while some isoforms contribute to hepatocellular carcinoma malignant phenotypes [37]. In MMTV-induced tumors with insertions at both genes, there may be synergistic activation by WNT and TCF7L2 leading to transformation.

We also investigated one additional CIS gene, *Arhgap18*, which is likely to be involved in signaling through pathways other than WNT. *Arhgap18* belongs to the human Rho GTPase activating protein (RhoGAP) family; approximately 80 RhoGAP proteins are known to be encoded in the human genome. RhoGAP proteins belong to the Ras superfamily, members of which participate in cell migration, intercellular adhesion, cytokinesis, proliferation, differentiation and apoptosis. The physiological role(s) of *Arhgap18* has not yet been elucidated, although one recent study has found that it plays a role in cell migration and shape [38].

While one of the initial goals of this analysis was to determine if MMTV(Y1Y2)-induced tumors showed a different pattern of integration sites than MMTV(HP) or MMTV(C3H) due to the loss of ITAM-mediated signaling, aside from the change in the ratio of *Wnt1* and *Fgf3* integrations that we previously reported [15], we did not detect any unique integration sites in these tumors, most likely due to the small number of tumors analyzed. However, pathway analysis of the integration sites in the two different sets of tumors indicated that there was a bias towards integration into genes involved in cell motility, angiogenesis and cell adhesion/invasiveness in the MMTV(Y1Y2) tumors (not shown).

What is the role of these novel MMTV CISs in human breast cancer? Interestingly, both *Antxr1* and *Ahrgap18* are over-expressed in human lobular and inflammatory breast cancer (Table 1). Additionally, some but not all studies have linked polymorphisms in *Tcf7l2* with increased breast cancer risk and more metastatic disease [39], [40], [41]. Thus, our study confirms previous work showing that the analysis of MMTV integration sites is likely to lead to new insights into the human disease and perhaps can identify novel potential therapeutic targets for human breast cancer treatment 2,4.

Most MMTV-induced mammary tumors contain 10 or more proviral integrations and it is thought that MMTV-induced tumors arise when multiple integrations occur in a single cell [5], [42]. Here, we show that tumors with multiple MMTV integrations frequently target *Wnt* and *Fgf* family members, but that there are additional CISs in MMTV-induced tumors that also participate in oncogenesis. Our data suggest that there are more oncogenes yet to be discovered by analyzing MMTV CISs.

## Materials and Methods

### Mice

C3H/HeN MMTV+ and BALB/c mice were purchased from the Animal Program of the National Cancer Institute. BALB/c mice infected with the HP and HP-Y1Y2 viruses were previously described [7]. MMTV-infected mice were palpated weekly starting at 5 months of age and sacrificed when tumors were less than 1 cm in diameter. All mice were housed according to the policies of the Institutional Animal Care and Use Committee of the University of Pennsylvania.

### Cell lines and reagents

NMuMG and HC11 mouse mammary cell lines were purchased from the American Type Culture Collection (Rockville, MD, USA). NMuMG cells were grown in DMEM media with 5% FBS, 10 · g/ml insulin, and penicillin/streptomycin. HC11 cells were grown in DMEM supplemented with 5% FBS, 10 · g/ml insulin, 10 ng/ml EGF, and penicillin/streptomycin. All cell lines were cultured at a constant temperature of 37°C in a 5% CO_2_ humidified atmosphere. Mouse fibroblast L cells (control, Wnt3a, Wnt5a) were obtained from American Type Culture Collection (Rockville, MD, USA) and grown in DMEM media with 5% FBS, and penicillin/streptomycin. Conditioned media were collected at 4 days after plating, when the cells were still sub-confluent.

### Cloning of integration sites from mouse mammary tumors

Genomic DNA was extracted from tumors using either Trizol Reagent (Invitrogen, Inc., Carlsbad, CA) according to the manufacturer's instructions or Proteinase K/SDS lysis. Cloning was performed by modifying the method of Schroder [14]. Four µg of genomic DNA from each tumor was digested with TaqI, BfaI-, or [Nhe I + Avr II + Spe I]-digested DNA. Following digestion, linker DNA was ligated to the digested genomic DNA, the samples were purified with the Strataprep PCR purification kit (Stratagene, Inc., La Jolla, CA) according to the manufacturer's instructions. By using 3 sets of enzymes that cut at different sites and with different frequencies in the genome, we controlled, at least in part, for cloning biases inherent in this method (restriction sites too close or too far from the integration site, for example). After amplification, all DNA was also restricted with PstI, which eliminated any products arising from the 5′ LTR. Following Pst I digestion samples were purified with the StrataPrep PCR purification kit. Sequences were amplified using BD Advantage 2 Taq polymerase (Clontech, Inc. Mountain View, CA), with one primer that hybridized to the linker DNA and the other to the MMTV 3′ LTR. The PCR products were purified with the StrataPrep PCR purification kit and diluted 1∶200. The diluted PCR products were amplified again using Platinum Taq Polymerase (Invitrogen) and nested primers that hybridized to the linker DNA and the 3′ LTR. The PCR products were purified with the StrataPrep PCR purification kit then cloned using the pGEM-T Vector System I (Promega, Inc., Madison, WI) according to the manufacturer's instructions. To maximize the number of integration sites cloned, the procedure was repeated for each tumor DNA using either Bfa I or “NAS” (combination of Nhe I, Avr II, and Spe I simultaneously) in place of Taq I in the first step and linkers modified for use with these enzymes. All linker sequences are available upon request.

### Integration site analysis

Clones were sequenced by the Sequencing Facility of the University of Pennsylvania School of Medicine. Integration sites were identified using the BLAT feature in the Mouse Genome Browser Gateway against the February 2006 freeze (mm8) of the mouse genome sequence. Integration sites were judged to be authentic only if a match to the mouse genome: (i) contained the proper 3′ LTR and started within 3 bp from the junction with the MMTV 3′ terminal sequence (5′-CCCA-3′) (ii) extended over the length of the sequence with an average identity >98%; and (iii) yielded a unique best hit in the BLAT ranking. Identical sequences from different clones isolated from the same tumor were judged to represent multiple isolates of a single integration event in that tumor.

### RNA analysis

Total RNA was isolated from tumors or normal tissue using the Trizol Reagent (Invitrogen, Inc.) according to the manufacturer's instructions. Before the RT reactions, all RNA samples were treated with 1 U of DNAse I (amplification grade, Invitrogen) for 10 min at room temperature in order to eliminate genomic DNA contamination. RNA was used to generate cDNA using Superscript II Reverse Transcriptase (Invitrogen, Inc.) according to the manufacturer's instructions. Reactions without the RT enzyme were used as negative controls.

Radioactive PCR was performed using Platinum Taq Polymerase, ^32^P-ATP and the primer pairs shown in Table 4. The amplification conditions for all PCR reactions except for the mouse β-actin (ActB) primers consisted of initial denaturation at 94°C for 2 min, denaturation at 94°C for 30 s, annealing at 55°C or 50°C for 30 s and extension at 72°C for 30 s for 35 cycles. The amplification conditions for the ActB primers consisted of initial denaturation at 94°C for 2 min, denaturation at 94°C for 30 s, annealing at 50°C for 30 s and extension at 72°C for 30 s for 21 cycles. The RT-PCR products were resolved on 5% acrylamide or 3% NuSieve agarose gels. The gels were dried and scanned with a Molecular Dynamics Storm Phosphorimager. Bands were analyzed with Molecular Dynamics ImageQuant 5.2 software. Data are presented as relative expression after normalization to the signal obtained with the ActB primers. Quantitative real-time polymerase chain reaction was used for detecting *Ccnd*1, *Myc, Tfr*1 and Axin2 transcripts. All primer sequences are shown in Table 4. GAPDH was used as the endogenous control gene and all values were normalized to GAPDH. Relative quantification (RQ) using the comparative CT method (ΔΔCt method) was applied for gene expression level analyses. Results were analyzed with ABI 7900 HT sequence detection system (SDS) v2.3 software using relative quantification study (ΔΔCt study) according to the manufacturer's instructions.

**Table 4:** Primer sequences used in RT-PCR analysis.

Genes	Forward primer	Reverse primer
*Ccnd1*	CCTGTTACCTGATACCTCTGC	CGGTGGTGGGAGAAGAGAGTTC
*Myc*	CTCTGCCTCTGCCCGCGATCA	CGGTGGAGAAGTTGCCACC
*Tfr1*	TAGAGTTTGCTGACACC	CACATAGTGTTCATCTCGCC
*Axin2*	TGACTCTCCTTCCAGATCCCA	TGCCCACACTAGGCTGACA
*Antxr1*	AAATGGCCCACAGTAGATGC	CTTGATGGGCGAGTACCACT
*Arhgap18*	TCGCAGATATGGCCAATACA	GACCTTCTTGGCGATTTTCA
*Tcf7l2*	CGGCCAAGAGGCAAGATGGA	GAGCGATCCGTTGGGGAGGT
*Actin*	TGGAATCCTGTGGCATCCATGAAAC	CGCTCCTGGAGGATGGTGAT
*GAPDH*	CCCCTTCATTGACCTCAACTACA	CGCTCCTGGAGGATGGTGAT
*Wnt1*	AGAACCCGGGGATCCTGCAC	GGAGCAGTGGGGCAGTTCCA
*Wnt10*	GGATCCACAACAACAGGGTGGGACG	TGGAAACGACAGTGGCAGCGCTCC
*Wnt3A*	GAGGAATGGTCTCTCGGGAGTTTGCCG	GGTGCGGTCACGCGTCCCGAAGGAGC
*Wnt3*	CTGAGGGAACCTCCACCATC	GACAACCCGTGGCATTTACA
*Fgf3*	GGAGATTACTGCGGTGGAAGTGGG	CTTTTGTGTGCGGCGGGTCTTGAA
*Fgf4*	GACCGCCGCACCCAACGGCACGCGG	GATGCCCACGTTGCAGTAGAGCCG
*Fgf8*	GCCCGTTTTGGTTTGGCAGCTTGC	GGAGCCCTTGCGGGGCCGGCCCTTGCG

### Plasmids and retroviral transductions

Plasmids containing the cDNAs of *Wnt1*, *Prkaca*, *Arhgap18*, *Tcf7l2*, and *Antxr1* were obtained from Open Biosystems. The cDNAs were PCR-amplified, HA-tagged and subcloned into the pBabe retroviral expression vector. After subcloning, the sequence of all constructs was confirmed by DNA sequencing. The pBabe vectors were packaged into amphotropic MLV particles and used to infect NMuMG and HC11 mouse mammary cells. Two days post-infection, the cells were selected by puromycin treatment. After 10 days of puromycin selection the surviving cells were pooled. The pooled cells were used for all experiments.

### Western blots

Western blots were probed with anti-HA (Invitrogen, Inc.) and anti-active β-catenin (Millipore, Billerica, MA) antibodies. The species-appropriate horseradish peroxidase-conjugated secondary antibody was used, followed by detection with ECL reagents (Amersham Biosciences, Inc.). For nuclear extraction, cells were incubated with hypotonic solution (25 mM KCl, 2 mM MgCl, 10 mM Hepes) for 30 min, and lysed with hypotonic solution containing 0.1% Triton X100. Nuclei were pelleted and dissolved in RIFA buffer. Anti-Lamin B (Santa Cruz Biotechnology, Inc., Santa Cruz, CA) was used as a nuclear specific internal control.

### Cell growth and proliferation assays

Cells (1×104 /well) were plated in 96-well plates and grown for 4 days in 2% or 5% FBS. At day 1, 3, and 4, the cells were incubated with 10 µl of MTT at 5 mg/ml (Sigma Chemical Co., St Louis, MO, USA) for 1 h at 37°C. 0.04 N HCl in isopropanol was added and the absorbance was measured in a microplate reader at a wavelength of 570 nm. Cell proliferation was detected by APC-BrDU Flow Kit (BD Biosciences, San Jose, CA). Cells (5×105 /well) were plated on 10 cm dishes. At 48 h after the initial seeding of the cells, 10 µM of BrdU was added to culture medium and incubated for 30 min. Cells were resuspended in binding buffer containing APC-conjugated anti-BrdU and 7-AAD solution. Cell suspensions were incubated for 15 minutes at room temperature and immediately analyzed in a flow cytometer (BD FACSCalibur, BD Biosciences, San Jose, CA). Data was analyzed by FlowJo (Tree Star, Inc., Ashland, OR).

### 3D Morphogenesis

Cells were plated as single cell suspensions on growth factor reduced Matrigel (BD BioSciences) using the overlay method [43]. Cells were maintained in culture for 2 wk changing medium once every 3 d. At 10 day after culture the matrigels containing acini were embedded in OCT medium (Triangle Medical Sciences, Durham, NC) and 10-µm-thick frozen sections were obtained. Samples were stained with hematoxylin and eosin (Sigma-Aldrich) to identify the presence of lumen in the acinar structures.

### Statistical analyses

GraphPad Prism version 5.00 for Windows (GraphPad Software, San Diego CA) was used in all statistical analyses. The cell growth data was analyzed using two-way analysis of variance (ANOVA) with Dunnett's post hoc test. All other data were analyzed using Student's t-test.

### Ethics Statement

All mice were housed according to the policies of the Institutional Animal Care and Use Committee (IACUC) of the University of Pennsylvania. The experiments performed with mice in this study were approved by this committee (IACUC protocol #801594).
